# Case Report: A novel *PTHLH* nonsense variant in a mother–son pair with brachydactyly type E and short stature, with a genotype–stature review

**DOI:** 10.3389/fendo.2026.1875896

**Published:** 2026-07-17

**Authors:** Hui Huang, Binyang Zhu, Zaisheng Wang, Zhuqiang Wu, Jinqiu Rao, Yu Yang, Xiangyu Xiong

**Affiliations:** 1 Jiangxi Provincial Key Laboratory of Child Development and Genetics, Jiangxi Provincial Children’s Hospital, The Affiliated Children’s Hospital of Nanchang Medical College, Nanchang, China; 2 Department of Child Health Care, Xiushui County Maternal and Child Health Hospital, Jiujiang, China; 3 Department of Radiology, Jiangxi Provincial Children’s Hospital, The Affiliated Children’s Hospital of Nanchang Medical College, Nanchang, China; 4 Medical Service Department, MyGenostics, Beijing, China; 5 Department of Endocrinology, Metabolism and Genetics, Jiangxi Provincial Children’s Hospital, The Affiliated Children’s Hospital of Nanchang Medical College, Nanchang, China

**Keywords:** brachydactyly type E, *PTHLH*, PTHrP, short stature, whole-exome sequencing

## Abstract

**Background:**

Brachydactyly type E is a rare skeletal disorder characterized by variable shortening of the metacarpals and/or metatarsals. Heterozygous pathogenic variants in *PTHLH*, which encodes parathyroid hormone-related protein, are an established cause of autosomal dominant brachydactyly type E and are variably associated with short stature.

**Case presentation:**

We report a mother-son pair with brachydactyly type E and short stature caused by a novel heterozygous nonsense variant in *PTHLH*. The proband, a boy aged 7 years and 8 months, presented with short stature, brachydactyly involving both hands and feet, mild global developmental delay, and subtle craniofacial features. Radiographs showed generalized shortening of the metacarpals, metatarsals, and phalanges, premature epiphyseal fusion, and metaphyseal widening. His mother had marked adult short stature and brachydactyly, with more prominent shortening of the third to fifth digits. Biochemical and endocrine evaluation revealed no evidence of hormone resistance or major metabolic abnormalities. Trio whole-exome sequencing identified a novel heterozygous nonsense variant in *PTHLH*(NM_198965.2) c.82G>T, p.(Glu28Ter), and Sanger sequencing confirmed segregation with the affected mother. Our pooled descriptive review of previously published individual-level cases showed that short stature is common but incompletely penetrant in *PTHLH*-associated brachydactyly type E. Predicted loss-of-function variants, especially early truncating, frameshift, and signal peptide/initiation-region variants, were more often reported with growth impairment, whereas recurrent variants and familial cases showed marked inter- and intrafamilial variability.

**Conclusion:**

This report expands the mutational spectrum of *PTHLH* and supports the role of *PTHLH* haploinsufficiency in impaired skeletal growth. This family further illustrates that *PTHLH*-associated brachydactyly type E can present with generalized involvement of the metacarpals, metatarsals, and phalanges. Variant consequence and protein location may help clinical risk stratification but are insufficient for reliable individual-level prediction of short stature. The molecular diagnosis also informed management by supporting longitudinal growth monitoring rather than recombinant growth hormone therapy in the absence of GH-IGF-1 axis abnormalities. Recognition of brachydactyly with short stature and normal biochemical findings should prompt consideration of *PTHLH*-related disease and molecular testing.

## 1 Introduction

Brachydactyly type E (BDE2; OMIM #613382) is a rare congenital skeletal disorder characterized by variable shortening of the metacarpals and/or metatarsals, often with involvement of the phalanges (1). It is genetically heterogeneous and may occur as an isolated skeletal trait or as part of a syndromic phenotype. Heterozygous pathogenic variants in *PTHLH*, which encodes parathyroid hormone-related protein, are a recognized cause of autosomal dominant *PTHLH*-associated brachydactyly type E, also referred to as BDE2.

Parathyroid hormone-related protein plays an essential role in endochondral ossification and longitudinal bone growth (2). In the growth plate, PTHrP interacts with the Indian hedgehog signaling pathway to regulate the balance between proliferating and hypertrophic chondrocytes. By delaying hypertrophic differentiation, PTHrP helps maintain the proliferative zone of the growth plate and prevents premature epiphyseal maturation. Disruption of this regulatory pathway may therefore lead to accelerated chondrocyte maturation, premature epiphyseal fusion, shortening of the metacarpals and metatarsals, and impaired linear growth.

The clinical spectrum of *PTHLH*-associated brachydactyly type E is broad. In addition to shortening of the metacarpals and metatarsals, affected individuals may present with short stature, disproportionate growth, cone-shaped epiphyses, dental anomalies, craniofacial features, neurodevelopmental findings, mammary abnormalities, thyroid abnormalities, obesity, or other systemic manifestations. However, these features show marked inter- and intrafamilial variability. In particular, short stature has been reported in many but not all affected individuals, and whether stature impairment is related to variant consequence or protein region remains incompletely defined.

Although *PTHLH* loss of function is an established mechanism underlying brachydactyly type E, fewer than 20 distinct *PTHLH* variants associated with this condition have been reported. Most published reports describe single individuals or small families, and height data are heterogeneous: some studies provide height standard deviation scores or percentiles, whereas others only state whether short stature was present. This limits the ability to determine whether specific variant classes, such as early truncating, frameshift, splice-altering, or signal peptide variants, are more consistently associated with growth impairment.

Here, we report a mother–son pair with brachydactyly type E and short stature caused by a novel heterozygous nonsense variant in *PTHLH*, NM_198965.2:c.82G>T, p.(Glu28Ter). We describe the clinical, radiographic, endocrine, and molecular findings in this family and perform a pooled descriptive review of previously published individual-level cases. In particular, we focused on whether short stature is related to *PTHLH* variant consequence and protein region, while recognizing the descriptive nature of the available literature.

## 2 Case presentation

### 2.1 Proband

The proband was a 7-year-8-month-old boy who was referred for evaluation of short stature. Growth retardation and mild global developmental delay had been noted since infancy and had persisted for more than 7 years. There was no history of seizures, fractures, or other major chronic medical conditions. Birth weight and birth length were unavailable. The parents were non-consanguineous. The mid-parental target height was approximately 162 cm.

**Figure 1:**
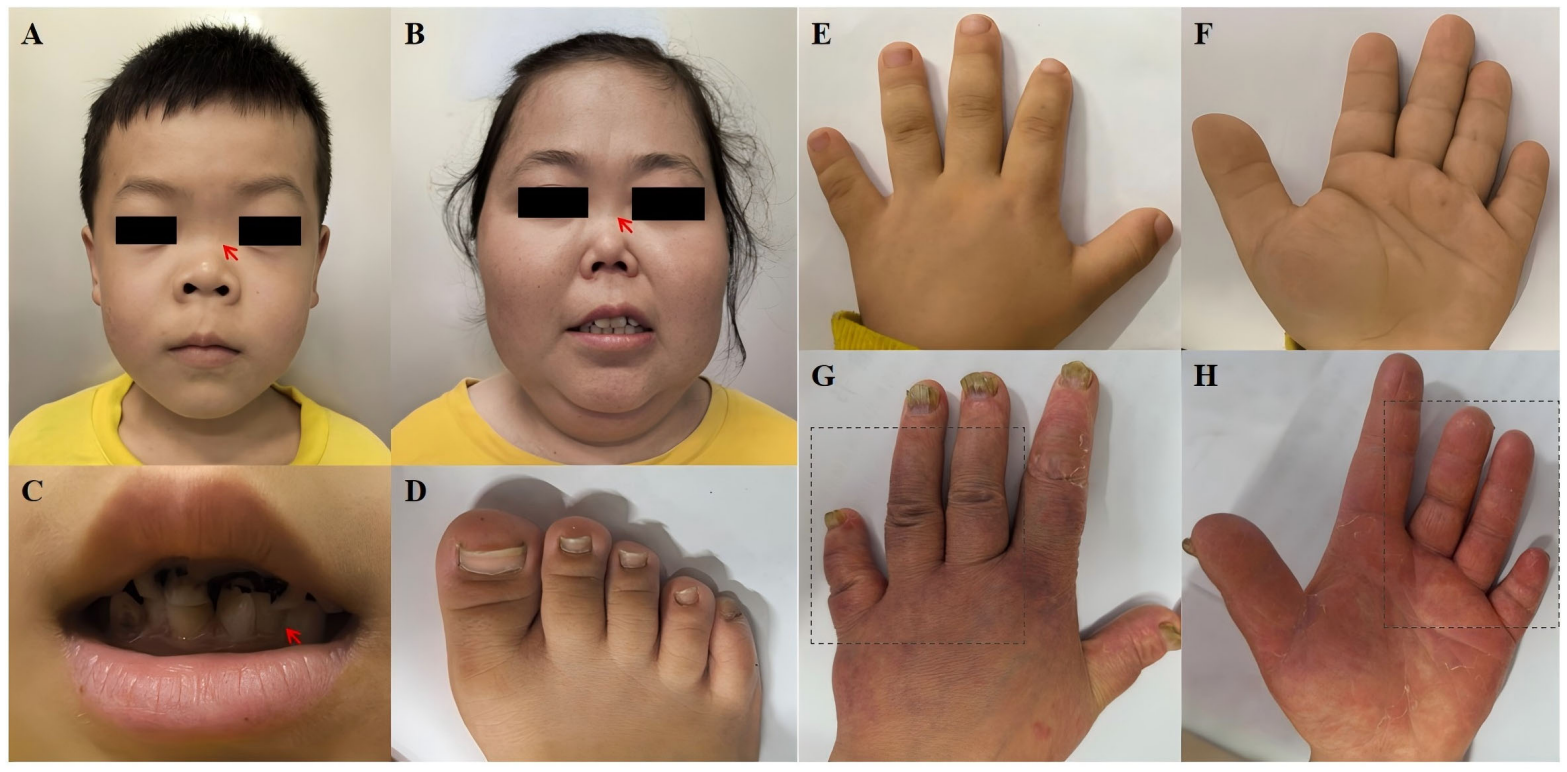
Clinical manifestations associated with the *PTHLH* c.82G>T (p.Glu28Ter) variant. **(A)** Frontal view of the proband showing a depressed nasal bridge. **(B)** Facial features of the affected mother with a similar craniofacial configuration. **(C)** Intraoral view demonstrating dental crowding and caries in the proband. **(D)** Shortened toes in the proband. **(E, F)** Dorsal and palmar views of the proband’s hands demonstrating generalized brachydactyly. **(G, H)** Dorsal and palmar views of the mother’s hands showing more prominent digital shortening, especially of the third to fifth digits.

At presentation, his height was 117 cm (−2.09 SDS), weight was 18 kg (−2.56 SDS), and body mass index (BMI) was 13.15 kg/m² (−1.87 SDS). Blood pressure was 100/70 mmHg.Head circumference was 51 cm, with no evidence of microcephaly or macrocephaly. Additional anthropometric assessment showed an upper segment of 61 cm and a lower segment of 56 cm, giving an upper-to-lower segment ratio of 1.09. The upper-to-lower segment ratio was within the expected range for age. His arm span was 115 cm, and arm span minus height was -2 cm. Physical examination showed short stature, small hands and feet, and brachydactyly involving both the hands and feet. Craniofacial features included a depressed nasal bridge, short philtrum, and short neck (**Figures 1A, C, D–F**). Dental crowding and caries were also noted. No scoliosis, chest wall deformity, or genital abnormality was observed.

Developmental assessment using the Gesell Developmental Schedules demonstrated mild global developmental delay, with developmental quotients of 62 in adaptive behavior, 66 in gross motor function, 72 in fine motor function, 56 in language, and 64 in personal-social function. Before this evaluation, he had not received systematic rehabilitation, physical therapy, or speech therapy. He attended a regular first-grade primary school. According to the family, his teacher reported poor academic performance.

### 2.2 Laboratory and radiographic findings

Biochemical and endocrine evaluation showed no major abnormalities.IGF-1 and IGFBP-3 were within age- and sex-specific reference ranges; no biochemical evidence of thyroid dysfunction, calcium-phosphate metabolism disorder, or hormone resistance was observed.

**Figure 2:**
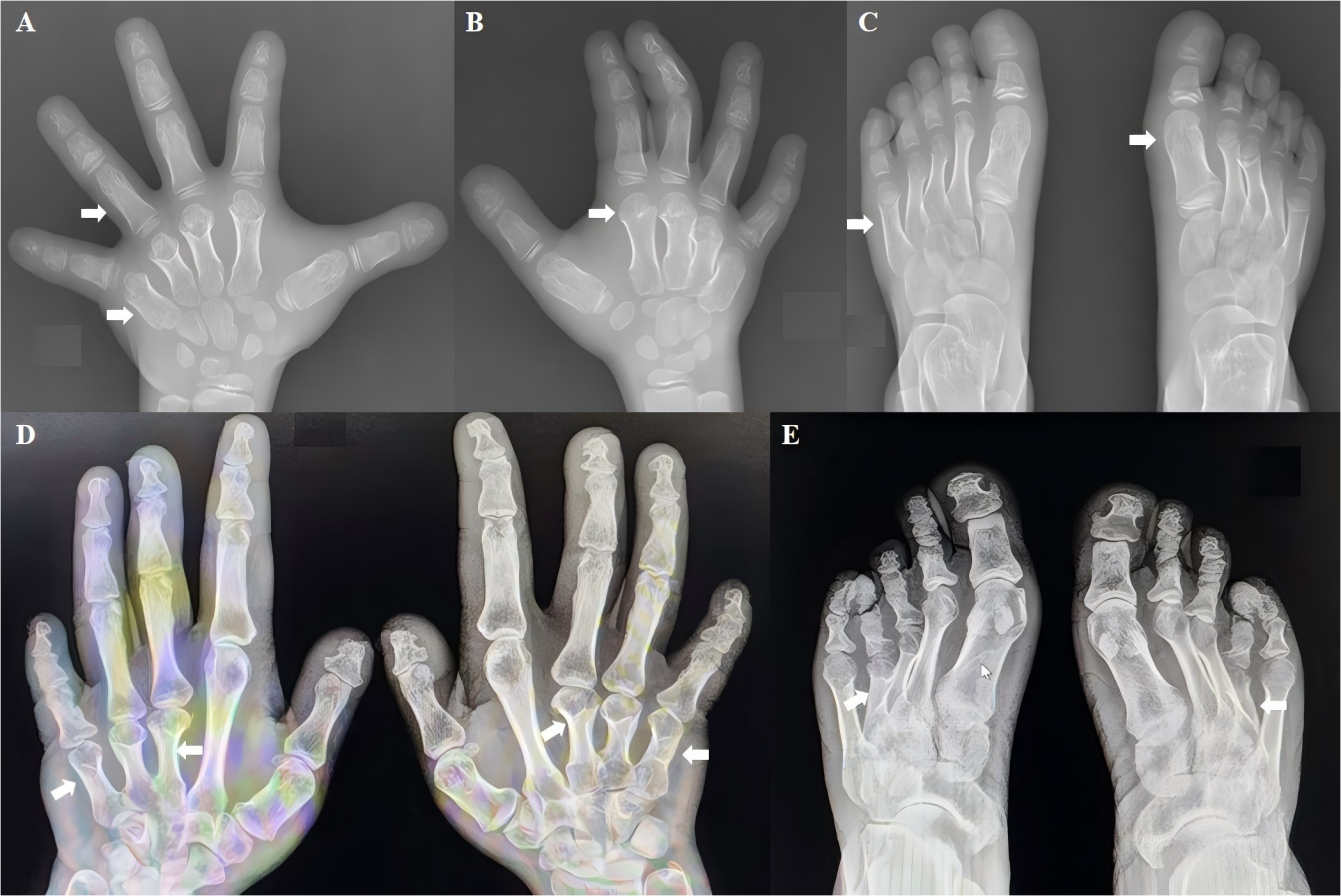
Radiographic features of *PTHLH*-related BDE2 in the proband and his mother. **(A)** Left hand radiograph of the proband demonstrating generalized shortening of the metacarpals and phalanges. **(B)** Evidence of premature epiphyseal fusion and metaphyseal widening in the proband. **(C)** Bilateral foot radiographs of the proband revealing diffuse shortening of the metatarsals and phalanges. **(D)** Bilateral hand radiographs of the mother showing marked shortening of metacarpals III–V and cone-shaped epiphyses. **(E)** Bilateral foot radiographs of the mother demonstrating metatarsal and phalangeal shortening.

Radiographs showed generalized shortening of metacarpals I–V and the corresponding phalanges, rather than predominant involvement of the fourth and fifth rays (**Figures 2A–C**). Premature epiphyseal fusion and metaphyseal widening were observed in the metacarpals. Foot radiographs showed diffuse shortening of the metatarsals and phalanges. Chest, pelvic, and spinal radiographs were unremarkable. Bone age, assessed by the Greulich–Pyle method, was 7 years at a chronological age of 7 years and 8 months.

### 2.3 Family history and maternal phenotype

Family history revealed similar skeletal findings in the patient’s mother. She had short stature, with an adult height of 140 cm (-3.81 SDS), and brachydactyly affecting both hands and feet. Digital shortening was more pronounced in the third to fifth digits (**Figures 1G, H**). Her weight was 60 kg (+1.12 SDS), and BMI was 30.61 kg/m², consistent with obesity. Additional anthropometric assessment showed an upper segment of 72 cm and a lower segment of 68 cm, corresponding to an upper-to-lower segment ratio of 1.05. Her arm span was 138.5 cm, and arm span minus height was -1.5 cm.

She also showed craniofacial features similar to those of the proband (**Figure 1B**). Radiographs of the mother showed marked shortening of metacarpals III-V with cone-shaped epiphyses (**Figure 2D**). Foot radiographs also demonstrated shortening of the metatarsals and phalanges(**Figure 2E**).On physical examination, the mother had no obvious dental anomalies, mammary abnormalities, or thyroid enlargement. Further laboratory evaluation for thyroid function, glucose and lipid metabolism, and insulin resistance was recommended but declined by the family.

According to the family’s report, the maternal grandparents had no apparent short stature or hand/foot abnormalities, but both had died before this study and were unavailable for clinical examination or genetic testing. The mother had no siblings; therefore, further segregation analysis in maternal relatives could not be performed. The father’s height was 171 cm (−0.21 SDS), and no comparable skeletal abnormalities were identified in him.

### 2.4 Genetic findings

**Figure 3:**
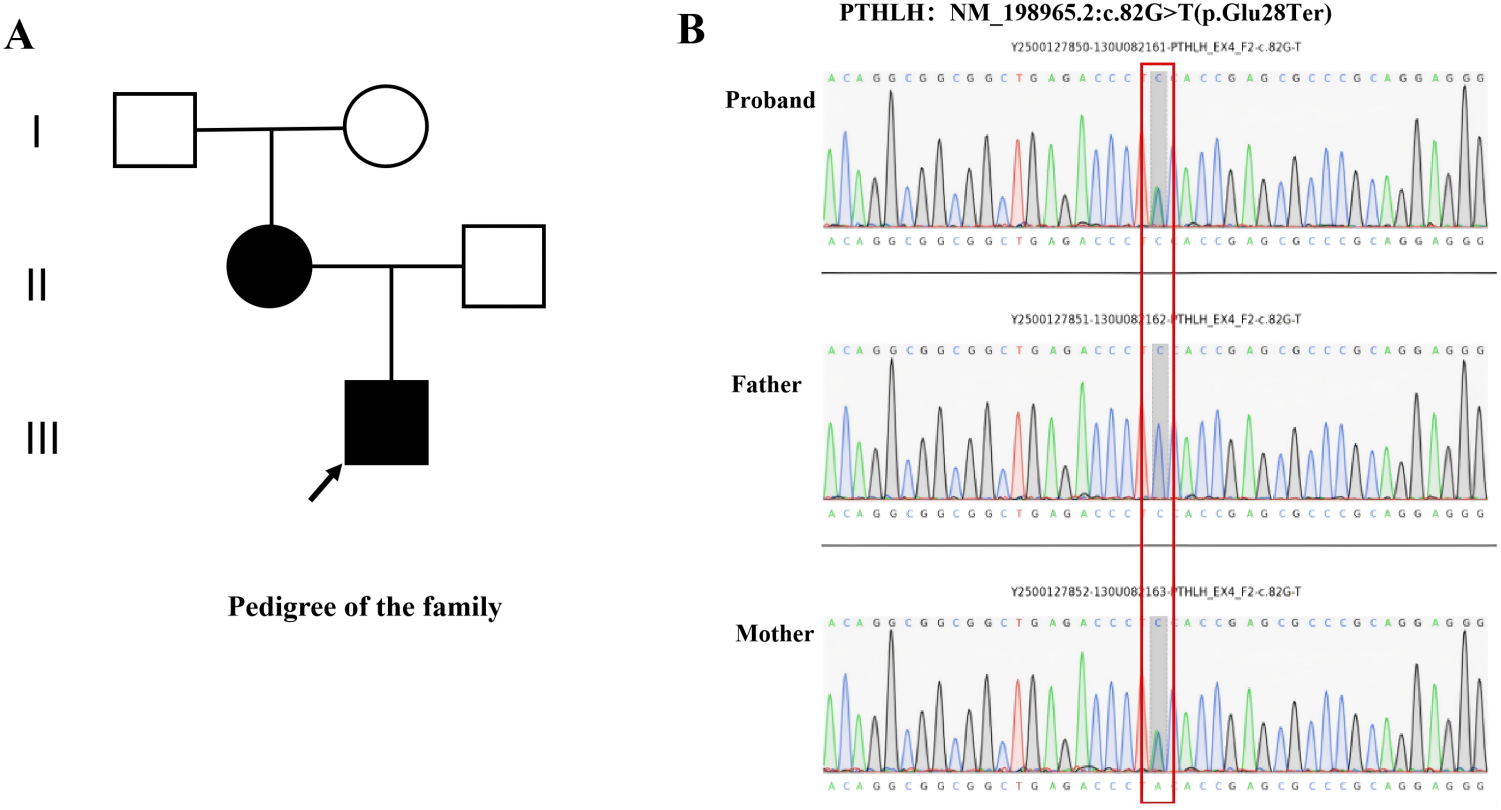
Pedigree analysis and Sanger sequencing validation of the *PTHLH* c.82G>T (p.Glu28Ter) variant. **(A)** Pedigree of the family. Filled symbols indicate affected individuals, and the proband is indicated by an arrow. **(B)** Sanger sequencing chromatograms showing a heterozygous c.82G>T substitution in the proband and the affected mother, whereas the father carries the wild-type allele. The variant position is highlighted.

Trio whole-exome sequencing identified a novel heterozygous nonsense variant in *PTHLH*, NM_198965.2:c.82G>T, p.(Glu28Ter), corresponding to chr12:27969413C>A in GRCh38.This variant is located in exon 4 of *PTHLH*. Sanger sequencing confirmed the presence of the variant in both the proband and his affected mother, whereas the unaffected father did not carry the variant (**Figure 3**). This segregation pattern supported autosomal dominant inheritance.

The variant was classified as pathogenic according to the ACMG/AMP guidelines (3) based on PVS1, PM2_Supporting, and PP4. PVS1 was applied because *PTHLH* is an established haploinsufficient gene and loss of function is a known disease mechanism for *PTHLH*-associated brachydactyly type E (4). PM2_Supporting was applied because the variant is absent from gnomAD v4.0. PP4 was applied because the patient’s phenotype, including brachydactyly type E, short stature, characteristic radiographic findings, and normal biochemical evaluation, is highly specific for *PTHLH*-associated disease (5). The variant has been submitted to ClinVar as a pathogenic variant under accession number SCV007602660.

### 2.5 Summary of published *PTHLH*-associated BDE cases

A structured literature search was performed in PubMed and Web of Science from database inception to February 2026. Search terms included “*PTHLH*,” “PTHrP,” “brachydactyly type E,” “BDE2,” “short stature,” “metacarpal shortening,” and “metatarsal shortening.” A total of 74 records were identified through database searching (PubMed, n=34; Web of Science, n=40). After removal of duplicates (n=32), 42 unique records remained for title and abstract screening. Of these, 21 were excluded. Twenty-one full-text articles were assessed for eligibility, and 10 were excluded for the following reasons: non-*PTHLH* etiology (n=6), structural variants including duplications or microdeletions (n=2), non-human study (n=1), and lack of extractable individual-level clinical data (n=1). Ultimately, 11 studies were included in the qualitative synthesis (**Table 1**; **Supplementary Table 1**).

**Table 1:** Summary of short stature in *PTHLH*-associated brachydactyly type E.

No.	Study/report	variant	Variant type	Protein region/location	N	Short stature, n/N assessed	Key height data
1	Elli 2022	c.2T>C, p.(Met1Thr)	Start loss	Start codon/signal peptide; Exon 4	1	1/1	Short stature described; exact height NR
2	Scheffer-Rath 2023	c.25T>C, p.(Trp9Arg)	missense	Signal peptide; Exon 4	4	3/4	Birth length -2.53 to -2.22 SDS; childhood height -2.3 to -1.35 SDS; maternal adult height -3.4 SDS
3	Wang 2015	c.44T>G, p.(Leu15Arg)	missense	Signal peptide; Exon 4	7	7/7	All affected individuals reported with height < -2 SD; individual height values NR
4	Thomas-Teinturier 2016	c.47_101 + 73del128, p.?	splicing	N-terminal coding region/splice donor; Exon 4–intron 4 junction	2	1/2	Proband 135.5 cm at 11.5 y, -1.5 SDS; mother adult height 147 cm, -3 SDS
5	Present	c.82G>T, p.(Glu28Ter)	nonsense	Early N-terminal region; Exon 4	2	2/2	Proband -2.09 SDS at 7 y 8 mo; mother adult height -3.81 SDS
6	Thomas-Teinturier 2016	c.101 + 3_101 + 6delAAGT, p.?	splicing	Splice donor region; Intron 4	1	0/1	+0.5 SDS at 12 y; predicted final height 156 cm (-1 SDS), target height 164 cm
7	Reyes 2019	c.102-3A>G, p.?	splicing	Splice acceptor region; Intron 4	5	2/5	Adult height -1.9 to -0.5 SDS; twin sons -0.9 and -0.5 SDS at 18 y/adult height
8	Fu 2019	c.125A>C, p.(Gln42Pro)	missense	N-terminal PTH-like domain; Exon 5	4	0/1; 3 NR	Proband 155.1 cm at 12 y, 50th percentile; mother, maternal uncle, and sister height NR
9	Klopocki 2010	c.131T>C, p.(Leu44Pro)	missense	N-terminal PTH-like domain; Exon 5	1	0/1	137 cm at 9 y 2 mo, +0.6 SDS
10	Sun 2024	c.146dupA, p.(Ser50ValfsTer22)	frameshift	N-terminal PTH-like domain; Exon 5	5	5/5	Proband 150 cm at 17 y; confirmed carriers 148–160 cm, reported as short stature/approximately -2 SD
11	Pereda 2017	c.166C>T, p.(Arg56Ter)	nonsense	N-terminal PTH-like domain; Exon 5	1	0/1	137.9 cm at 9 y 10 mo, 0 SDS; 148.7 cm at 12 y, +0.4 SDS; target height 164 cm
12	Jamsheer 2016	c.166C>T, p.(Arg56Ter)	nonsense	N-terminal PTH-like domain; Exon 5	1	0/1	158 cm at 13 y, 25th-50th percentile
13	Elli 2022	c.166C>T, p.(Arg56Ter)	nonsense	N-terminal PTH-like domain; Exon 5	1	1/1	Short stature described; exact height NR
14	Bae 2018	c.169C>T, p.(Arg57Ter)	nonsense	N-terminal PTH-like domain; Exon 5	3	0/2; 1 NR	Proband 175 cm at 31 y; mother not short, exact height NR; maternal grandmother height NR
15	Klopocki 2010	c.179T>C, p.(Leu60Pro)	missense	N-terminal PTH-like domain; Exon 5	1	1/1	41-year-old female; -2.97 SDS
16	Jamsheer 2016	c.258delC, p.(Asn87ThrfsTer18)	frameshift	Mid-region; Exon 5	3	2/3	Proband: <3rd to 3rd-10th percentile at 4.5–6 y; sister: 3rd percentile at 9 y; father 184 cm at 47 y, 75th-90th percentile
17	Elli 2022	c.299del, p.(Glu100GlyfsTer5)	frameshift	Mid-region/NLS-containing domain; Exon 5	1	1/1	Short stature described; exact height NR
18	Klopocki 2010	c.358A>T, p.(Lys120Ter)	nonsense	Mid-region; Exon 5	1	1/1	Short stature described; exact height NR
19	Klopocki 2010	c.532A>G,p.(Ter178GlyextTer53)	stop-loss	Stop codon/C-terminal extension; Exon 5	3	2/2; 1 NR	Proband -2.4 SDS at 14 y; maternal grandfather -3.6 SDS; mother height NR

Variants are described according to HGVS nomenclature using the *PTHLH* reference transcript NM_198965.2. Exon and intron locations were harmonized according to this transcript and may differ from the numbering used in the original reports. Protein regions were assigned according to reported PTHrP precursor and bioactive peptide domains. N indicates the number of reported individuals summarized for each variant or report. In “Short stature, n/N assessed,” n/N refers to individuals with available stature assessment; individuals without available height data are indicated as NR. Short stature was defined as height ≤ −2 SDS or as short stature explicitly described in the original report when SDS or percentile data were unavailable. Height data are presented with age at assessment whenever available; adult height is specified when reported. NR, not reported; SDS, standard deviation score; BDE, brachydactyly type E. Detailed individual-level height data and clinical features are provided in **Supplementary Table 1**.

Extracted data included nucleotide and protein variants, variant consequence, protein region, number of reported individuals, height data, stature category, BDE skeletal phenotype, and additional clinical features. Because of the small number of reports, familial clustering, and heterogeneous reporting of height data, the analysis was descriptive and exploratory; no inferential genotype–phenotype statistics were performed.

Together with the present family, the pooled dataset included 19 variant- or report-level entries and 47 affected individuals. Stature information was available for 42 individuals. Twenty of 42 individuals (47.6%) had height ≤ −2 SDS and were classified as having short stature. When individuals explicitly described as having short stature in the original reports but without sufficient numerical height data were also included, 31 of 42 individuals (73.8%) were classified as having short stature or reduced stature. These findings indicate that short stature is common but incompletely penetrant in *PTHLH*-associated brachydactyly type E.

Predicted loss-of-function variants, particularly early truncating variants, frameshift variants, and variants affecting the signal peptide or initiation region, were more often reported with growth impairment. However, because of the small number of cases, familial clustering, and heterogeneous reporting of height data, this pattern was interpreted descriptively rather than statistically. The c.166C>T, p.(Arg56Ter) variant, which has been reported in several unrelated cases or families, showed discordant stature phenotypes, and several familial cases demonstrated marked intrafamilial variability. These findings indicate that variant consequence and protein region may provide useful risk-stratification information but are insufficient for reliable individual-level prediction of short stature.

### 2.6 Patient perspective

The family’s main concern at presentation was the proband’s short stature and small hands and feet. Before molecular diagnosis, the family hoped that clarifying the etiology of short stature might guide treatment, including whether recombinant growth hormone therapy was appropriate. After *PTHLH*-associated brachydactyly type E was diagnosed, the family was counseled that the proband’s short stature was more likely related to altered growth-plate regulation than to a primary GH-IGF-1 axis defect. Given the absence of clear GH deficiency and the lack of evidence supporting GH therapy specifically for *PTHLH*-associated BDE, recombinant growth hormone was not initiated. The genetic diagnosis helped the family understand the shared mother-son phenotype, clarified recurrence risk, and guided long-term growth monitoring and reproductive counseling.

## 3 Discussion

In this study, we report a mother–son pair with brachydactyly type E and short stature caused by a novel heterozygous nonsense variant in *PTHLH* c.82G>T. The proband presented with short stature, generalized brachydactyly involving both hands and feet, premature epiphyseal fusion, metaphyseal widening, mild global developmental delay, and subtle craniofacial features. His mother also had marked adult short stature and brachydactyly, with more prominent involvement of the third to fifth digits. The variant segregated with the phenotype in the family and was absent in the unaffected father, supporting autosomal dominant inheritance. The clinical, radiographic, biochemical, and molecular findings are consistent with *PTHLH*-associated brachydactyly type E (1).

PTHrP plays a central role in endochondral ossification and longitudinal bone growth (6). In the growth plate, PTHrP interacts with the Indian hedgehog signaling pathway to maintain proliferating chondrocytes and delay hypertrophic differentiation. Disruption of this regulatory loop can accelerate chondrocyte maturation, promote premature epiphyseal fusion, and impair longitudinal bone growth (2). The radiographic findings in the present proband, including generalized shortening of the metacarpals, metatarsals, and phalanges, premature epiphyseal fusion, and metaphyseal widening, provide phenotypic support for this mechanism. The affected mother showed marked shortening of metacarpals III–V with cone-shaped epiphyses, further supporting a growth-plate disorder consistent with PTHrP haploinsufficiency.

In this case, the *PTHLH* p.(Glu28Ter) is located in the early N-terminal region of PTHrP and is predicted to introduce a premature termination codon. Because loss of function is an established mechanism for *PTHLH*-associated brachydactyly type E, this early truncating variant is likely to result in severe reduction of functional PTHrP, potentially through nonsense-mediated mRNA decay. Both affected family members had short stature, suggesting that this early nonsense variant may have a substantial effect on linear growth in this family.

Our structured literature review further supports that short stature is a frequent but incompletely penetrant feature of *PTHLH*-associated brachydactyly type E (7–9). Across previously published cases and the present family, short stature or reduced stature was more often reported in individuals carrying predicted loss-of-function variants, particularly early truncating variants, frameshift variants, and variants affecting the signal peptide or initiation region. Variants in the signal peptide region (8, 9), such as p.(Trp9Arg) and p.(Leu15Arg), were frequently associated with growth impairment, suggesting that altered PTHrP translation, processing, or secretion may contribute to impaired skeletal growth. Similarly, the present p.(Glu28Ter) variant and the previously reported p.(Ser50ValfsTer22) frameshift variant were associated with short stature in multiple affected individuals.

Nevertheless, the available data do not support a deterministic genotype-stature correlation. Several variants located in the N-terminal PTH-like domain showed discordant stature phenotypes. The p.(Arg56Ter) variant, which has been reported in three unrelated cases/families (7, 10, 11), is particularly informative, carriers have been described with normal stature, predicted final height below the target height, or clinically reported short stature.

Notably, patients carrying other pathogenic *PTHLH* variants have also been reported with brachydactyly type E but normal stature, further supporting the variable expressivity of stature phenotypes among *PTHLH* variant carriers (12–14) Familial cases also demonstrated marked intrafamilial variability (15). For example, c.47_101** + **73del128 (16) was associated with adult height below -2 SDS in the affected mother but only reduced stature in the proband; c.258delC (10), was associated with reduced stature in the proband and her sister but normal adult height in their father; and c.102-3A>G (17) showed stature ranging from borderline short stature to normal adult height within the same family. These findings indicate that variant consequence and protein location may provide useful risk-stratification information, but they are insufficient for reliable prediction of final height in an individual patient.

The clinical severity of *PTHLH*-associated brachydactyly type E also appears to extend beyond stature (7, 13, 16). In the individual-level summary, some patients had craniofacial features, neurodevelopmental findings, dental anomalies, mammary abnormalities, thyroid abnormalities, obesity, insulin resistance, or other nonskeletal manifestations (**Supplementary Table 1**). However, these features were inconsistently reported across studies, and their relationship with *PTHLH* haploinsufficiency remains uncertain. In the present family, the proband had mild global developmental delay and subtle craniofacial features, whereas the mother had short stature, obesity, and brachydactyly. Although these findings broaden the clinical context of the family, the current evidence is insufficient to establish robust genotype-specific associations for non-skeletal manifestations.

Clinically, recognition of the skeletal pattern is important. Children presenting with short stature and small hands or feet may initially be evaluated for endocrine disorders, especially pseudohypoparathyroidism, acrodysostosis, or other causes of Albright hereditary osteodystrophy-like phenotypes. In the present case, serum calcium, phosphorus, alkaline phosphatase, parathyroid hormone, thyroid function, IGF-1, and IGFBP-3 were within reference ranges, and no evidence of hormone resistance or major metabolic abnormality was identified. This combination of metacarpal/metatarsal shortening, normal calcium-phosphate metabolism, and familial skeletal findings should prompt consideration of *PTHLH*-associated brachydactyly type E. Molecular diagnosis is valuable not only for confirming the etiology but also for genetic counseling, because affected individuals have a 50% recurrence risk for offspring.

At present, there is no established evidence-based treatment for short stature specifically caused by *PTHLH*-associated BDE. Growth impairment in this disorder is thought to result primarily from altered PTHrP-mediated growth-plate regulation rather than from a primary GH-IGF-1 axis defect. Therefore, recombinant GH therapy cannot currently be recommended solely on the basis of a *PTHLH* variant. In the present patient, IGF-1 and IGFBP-3 were within reference ranges, and no clear evidence of GH deficiency was identified. After molecular diagnosis, recombinant GH treatment was not initiated; instead, longitudinal monitoring of growth velocity, bone age, pubertal development, and predicted adult height was recommended.

At present, no disease-specific therapy has been established for short stature caused by *PTHLH*-associated BDE. Growth impairment in this disorder is thought to result primarily from altered PTHrP-mediated growth-plate regulation rather than from a primary GH-IGF-1 axis defect. Moreover, to our knowledge, no published evidence has demonstrated the efficacy of recombinant GH therapy in patients with *PTHLH*-associated BDE. In the present patient, IGF-1 and IGFBP-3 were within reference ranges, bone age was not advanced, and no clear evidence of GH deficiency was identified. After discussion with the family, the uncertain benefit, treatment burden, and the family**’**s limited financial resources were also considered; therefore, recombinant GH therapy was not recommended. Subsequent clinical follow-up was advised according to standard pediatric endocrine practice. 

The present study has several limitations. First, the reported family includes only two affected individuals, and functional studies of the novel variant were not performed. Second, birth length and birth weight were unavailable for the proband, limiting assessment of prenatal growth. Third, the literature review was based mainly on case reports and small families with heterogeneous phenotypic documentation. Height SDS, adult height, bone age, and longitudinal growth data were unavailable or incomplete in many published cases. Fourth, related individuals within families cannot be treated as fully independent observations, and familial clustering may have influenced the apparent frequency of short stature for some recurrent or family-reported variants. Therefore, no inferential genotype–phenotype statistical analysis was performed. Our review should therefore be interpreted as a pooled descriptive analysis rather than a formal predictive model.

## 4 Conclusion

In summary, this study expands the mutational spectrum of *PTHLH* by identifying a novel early nonsense variant associated with brachydactyly type E and short stature in a mother-son pair. The literature review suggests that short stature is common but not obligatory in *PTHLH*-associated brachydactyly type E. Predicted loss-of-function variants, particularly early truncating, frameshift, and signal peptide/initiation-region variants, may be more often associated with growth impairment, but substantial inter- and intrafamilial variability precludes reliable individual-level prediction. Longitudinal growth monitoring, bone-age assessment, standardized phenotyping, and functional studies will be needed to clarify how different *PTHLH* variant classes influence stature and overall clinical severity.

## Data Availability

The variant data presented in this study are deposited in the ClinVar repository under accession number SCV007602660. All other data generated or analyzed during this study are included in the article and its **Supplementary Material**.
