# Supplementary material for: Case Report: A novel PTHLH nonsense variant in a mother–son pair with brachydactyly type E and short stature, with a genotype–stature review
Source: Front Endocrinol (Lausanne). 2026 Jul 17;17:1875896. doi: 10.3389/fendo.2026.1875896 (PMC13423629; doi:10.3389/fendo.2026.1875896)
Supplement: Supplementary file 1 [file Table1.docx]

**Supplementary Table S1. Clinical and molecular features of reported PTHLH-associated brachydactyly type E cases**

| **No.** | **Study / individual** | **variant** | **Variant Type** | **Location** | **Stature category** | **Height data, age at assessment** | **BDE skeletal phenotype** | **Craniofacial features** | **Neurodevelopment** | **Dental anomalies** | **Mammary abnormalities** | **Thyroid abnormalities** | **Other phenotypes** |
| --- | --- | --- | --- | --- | --- | --- | --- | --- | --- | --- | --- | --- | --- |
| 1 | Elli 2022 / Patient 1 | c.2T>C, p.(Met1Thr) | start-loss | Exon 4 | SS-described | NR | Brachymetacarpia, mainly 4th-5th; brachymetatarsia, mainly 4th; middle phalanges shortened | Absent / NR | Absent | NR | NA | Absent / NR | Rhizomelic shortening of upper and lower limbs; skull vault thickening |
| 2 | Scheffer-Rath 2023 / proband | c.25T>C, p.(Trp9Arg) | missense | Exon 4 | SS-SDS | Birth length 40 cm, -2.22 SDS; 91.4 cm at 3 y, -2.3 SDS; 149.4 cm at 13 y, -1.52 SDS | Short 4th metacarpal and short metatarsals | Present: frontal bossing, depressed nasal bridge | Present: speech/language delay; IQ 79 / learning disability | Absent: no oligodontia | NA | Absent / NR | Advanced bone age; disproportionate short stature |
| 3 | Scheffer-Rath 2023 / mother of proband | c.25T>C, p.(Trp9Arg) | missense | Exon 4 | SS-SDS | Adult height 149.5 cm, -3.4 SDS | Short 4th metacarpal of right hand; short 4th metatarsal of left foot | NR | Present: history of speech delay | Absent: no oligodontia | Present: unable to breastfeed; fertility treatment reported | Absent / NR | Back and fatigue complaints |
| 4 | Scheffer-Rath 2023 / younger brother of proband | c.25T>C, p.(Trp9Arg) | missense | Exon 4 | SS-SDS | Birth length 45 cm, -2.53 SDS; 85.5 cm at 2.3 y, -1.71 SDS | NR | NR | Present: speech/language delay at 2.3 y | Present: missing 1 upper and 1 lower lateral incisor | NA | Absent / NR | Feeding problem in early childhood |
| 5 | Scheffer-Rath 2023 / younger sister of proband | c.25T>C, p.(Trp9Arg) | missense | Exon 4 | SS-described | 68.5 cm at 9 mo, -1.35 SDS | NR | NR | Present: speech delay at 2 y | NR | NA | NR | Feeding problem in infancy |
| 6 | Wang 2015 / family report, 7 affected individuals | c.44T>G, p.(Leu15Arg) | missense | Exon 4 | SS-SDS | All seven affected individuals reported with height < -2 SD; individual height values NR | Bilateral severe generalized brachydactyly involving all metacarpals/metatarsals and phalanges | Present: round face; other facial features normal | Absent: intelligence normal | Absent: teeth normal | NR | Absent: thyroid testing normal | Normal bone/hormone laboratory results; functional evidence of impaired signal peptide cleavage |
| 7 | Thomas-Teinturier 2016 / proband | c.47_101+73del128, p.? | splicing | Exon 4-intron 4 junction | SS-described | Birth length 49 cm, -1 SDS; 135.5 cm at 11.5 y, -1.5 SDS | Shortening of 4th-5th metacarpals and 4th metatarsal; premature epiphyseal fusion | NR | Absent: normal intellectual development | Present: dental malpositions; no missing teeth | Present: poor/small breast development | Absent: normal TSH testing | Low apparent bone mineral density; reduced arm span |
| 8 | Thomas-Teinturier 2016 / mother of proband | c.47_101+73del128, p.? | splicing | Exon 4-intron 4 junction | SS-SDS | Adult height 147 cm, -3 SDS | Short 3rd-5th metacarpals, all distal phalanges, and 3rd-4th metatarsals | NR | Absent: normal intellectual development | Present: gingival masses; no oligodontia | Present: small breasts; able to breastfeed | Absent: normal TSH testing | Short forearms/lower legs; low apparent bone mineral density |
| 9 | Present study / proband | c.82G>T, p.(Glu28Ter) | nonsense | Exon 4 | SS-SDS | 117 cm at 7 y 8 mo, -2.09 SDS | Generalized shortening of metacarpals I-V and corresponding phalanges; diffuse shortening of metatarsals and phalanges | NR | NR | NR | NA | NR | Premature epiphyseal fusion and metaphyseal widening |
| 10 | Present study / mother of proband | c.82G>T, p.(Glu28Ter) | nonsense | Exon 4 | SS-SDS | Adult height 140 cm, -3.81 SDS | Marked shortening of metacarpals III-V with cone-shaped epiphyses; shortening of metatarsals and phalanges | NR | NR | NR | NR | NR | NR |
| 11 | Thomas-Teinturier 2016 / Patient 3 | c.101+3_101+6delAAGT, p.? | splicing | Intron 4 | Non-SS | +0.5 SDS at 12 y; predicted final height 156 cm (-1 SDS), target height 164 cm | Isolated shortening of 4th metacarpals and 4th metatarsals | NR | Absent: general development normal | Absent: dental development normal | Absent / normal pubertal breast development reported | Absent: normal TSH testing | Advanced bone age; premature growth arrest around 13 y |
| 12 | Reyes 2019 / maternal aunt of proband | c.102-3A>G, p.? | splicing | Intron 4 | SS-described | Adult height 151.0 cm, -1.9 SDS | Short hands and feet; shortening of multiple metacarpals and metatarsals | NR | NR | NR | NR | NR | NR |
| 13 | Reyes 2019 / mother of proband | c.102-3A>G, p.? | splicing | Intron 4 | SS-described | Adult height 155.0 cm, -1.3 SDS | Shortening of multiple metacarpals and metatarsals | NR | NR | NR | NR | NR | NR |
| 14 | Reyes 2019 / proband | c.102-3A>G, p.? | splicing | Intron 4 | Non-SS | Adult height 160.0 cm, -0.5 SDS | Shortening of the 3rd and/or 4th metacarpals | NR | NR | NR | NR | NR | NR |
| 15 | Reyes 2019 / twin son of proband | c.102-3A>G, p.? | splicing | Intron 4 | Non-SS | Birth length 41.0 cm; adult height 169.5 cm, -0.9 SDS | Shortening of the 4th metacarpals | NR | NR | NR | NR | NR | NR |
| 16 | Reyes 2019 / twin son of proband | c.102-3A>G, p.? | splicing | Intron 4 | Non-SS | Birth length 40.5 cm; adult height 172.5 cm, -0.5 SDS | Shortening of the 4th metacarpals | NR | NR | NR | NR | NR | NR |
| 17 | Fu 2019 / proband | c.125A>C, p.(Gln42Pro) | missense | Exon 5 | Non-SS | 155.1 cm at 12 y, 50th percentile | Short 3rd-5th metacarpals | NR | Normal intelligence reported | NR | NA | NR | Obesity; BMI 32.01 kg/m2 |
| 18 | Fu 2019 / mother of proband | c.125A>C, p.(Gln42Pro) | missense | Exon 5 | NR | NR | Similar brachydactyly phenotype reported | NR | NR | NR | NR | NR | NR |
| 19 | Fu 2019 / maternal uncle of proband | c.125A>C, p.(Gln42Pro) | missense | Exon 5 | NR | NR | Similar brachydactyly phenotype reported | NR | NR | NR | NA | NR | NR |
| 20 | Fu 2019 / younger sister of proband | c.125A>C, p.(Gln42Pro) | missense | Exon 5 | NR | NR | Similar brachydactyly phenotype reported | NR | NR | NR | NA | NR | NR |
| 21 | Klopocki 2010 / proband, family 3 | c.131T>C, p.(Leu44Pro) | missense | Exon 5 | Non-SS | 137 cm at 9 y 2 mo, +0.6 SDS | Short 3rd-5th metacarpals | NR | NR | Present: problems with tooth eruption / abnormal dentition | NR | NR | Prematurely fused abnormal metacarpal epiphyses |
| 22 | Sun 2024 / proband | c.146dupA, p.(Ser50ValfsTer22) | frameshift | Exon 5 | SS-described | 150 cm at 17 y; reported < -1 SD / short stature | Shortened metacarpals and phalanges of both hands; shortened 4th-5th toes/metatarsals | NR | NR | NR | NA | NR | NR |
| 23 | Sun 2024 / affected relative of proband | c.146dupA, p.(Ser50ValfsTer22) | frameshift | Exon 5 | SS-SDS | 149 cm at 32 y, -2 SD | Similar hand phenotype; right foot short 3rd-5th metatarsals; left foot short 3rd-4th metatarsals | NR | NR | NR | NR | NR | NR |
| 24 | Sun 2024 / affected relative of proband | c.146dupA, p.(Ser50ValfsTer22) | frameshift | Exon 5 | SS-SDS | 160 cm, -2 SD; age NR | Short 4th metacarpals in both hands | NR | NR | NR | NA | NR | NR |
| 25 | Sun 2024 / affected relative of proband | c.146dupA, p.(Ser50ValfsTer22) | frameshift | Exon 5 | SS-SDS | 148 cm, -2 SD; age NR | Bilateral shortened metacarpals and phalanges, especially middle phalanx of 3rd finger | NR | NR | NR | NR | NR | NR |
| 26 | Sun 2024 / affected relative of proband | c.146dupA, p.(Ser50ValfsTer22) | frameshift | Exon 5 | SS-SDS | 149 cm, -2 SD; age NR | Bilateral shortened metacarpals and phalanges | NR | NR | NR | NR | NR | NR |
| 27 | Pereda 2017 / proband | c.166C>T, p.(Arg56Ter) | nonsense | Exon 5 | Non-SS | Birth length 47 cm, -1.47 SDS; 137.9 cm at 9 y 10 mo, 0 SDS; 148.7 cm at 12 y, +0.4 SDS; target height 164 cm | Shortening of all metacarpals and distal phalanges of 1st and 3rd fingers; shortening of 3rd-5th metatarsals | Present: round face, long philtrum, short neck | NR | NR | NA | NR | Advanced bone age; mild overweight; premature pubarche/early menarche; paternal mosaicism |
| 28 | Jamsheer 2016 / sporadic proband | c.166C>T, p.(Arg56Ter) | nonsense | Exon 5 | Non-SS | 158 cm at 13 y, 25th-50th percentile | BDE of hands and feet; hand involvement most pronounced in fingers 2, 3, and 5; feet with short toes 3-5 | Present: craniofacial dysmorphism | Absent: intellectually normal | NR | NA | NR | De novo variant; normal bone age |
| 29 | Elli 2022 / Patient 2 | c.166C>T, p.(Arg56Ter) | nonsense | Exon 5 | SS-described | NR | Brachymetacarpia; dysmorphic 2nd phalanges II and V; brachymetatarsia III; toe brachyphalangy | Absent / NR | Absent | Present: delayed eruption of definitive molar teeth | NA | Absent / NR | Cafe-au-lait spots |
| 30 | Bae 2018 / proband | c.169C>T, p.(Arg57Ter) | nonsense | Exon 5 | Non-SS | 175 cm at 31 y | Shortening of 4th-5th metacarpals and 4th-5th metatarsals | NR | NR | Absent | NA | NR | NR |
| 31 | Bae 2018 / mother of proband | c.169C>T, p.(Arg57Ter) | nonsense | Exon 5 | Non-SS | Not short; exact height NR | Similar shortening of 4th-5th metacarpals and 4th-5th metatarsals | NR | NR | Absent / NR | Absent / NR | NR | NR |
| 32 | Bae 2018 / maternal grandmother of proband | c.169C>T, p.(Arg57Ter) | nonsense | Exon 5 | NR | Height NR; clinically affected relative reported, not molecularly confirmed | Similar hand/foot phenotype reported | NR | NR | NR | NR | NR | NR |
| 33 | Klopocki 2010 / proband, family 2 | c.179T>C, p.(Leu60Pro) | missense | Exon 5 | SS-SDS | 41-year-old female; -2.97 SDS | Short 3rd-5th metacarpals; short middle and distal phalanges of index and 5th fingers | Present: round face | NR | Present: problems with tooth eruption | NR | NR | Functional loss-of-function missense variant |
| 34 | Jamsheer 2016 / proband | c.258delC, p.(Asn87ThrfsTer18) | frameshift | Exon 5 | SS-described | 100 cm at 4.5 y, <3rd percentile; 109.5 cm at 6 y, 3rd-10th percentile | Generalized brachydactyly of hands and feet; hands most affected in digits 2 and 5; feet with short toes 1-4 | Present: facial dysmorphism | Absent: psychomotor development normal | NR | NA | NR | Delayed bone age; thoracic scoliosis; lumbar hyperlordosis |
| 35 | Jamsheer 2016 / sister of proband | c.258delC, p.(Asn87ThrfsTer18) | frameshift | Exon 5 | SS-described | 124 cm at 9 y, 3rd percentile | BDE of both hands and feet; hand shortening involving thumbs and 5th fingers; feet with short toes 3-5 | Present: facial dysmorphism | Absent: intellectual development normal | NR | NA | Present: hypothyroidism with elevated anti-TPO antibodies | Delayed bone age |
| 36 | Jamsheer 2016 / father of proband | c.258delC, p.(Asn87ThrfsTer18) | frameshift | Exon 5 | Non-SS | 184 cm at 47 y, 75th-90th percentile | Mild/discrete brachydactyly; short/stubby thumbs; mild shortening of post-axial rays in feet | Absent / NR | NR | NR | NA | NR | Mild phenotype |
| 37 | Elli 2022 / Patient 3 | c.299del, p.(Glu100GlyfsTer5) | frameshift | Exon 5 | SS-described | NR | Asymmetric brachymetacarpia: left 4th-5th, right 3rd-5th; bilateral brachyphalangy involving 1st-4th digits | Present: posterior plagiocephaly; Chiari malformation II | NR | Present: supernumerary tooth | NA | Absent / NR | Obesity; insulin resistance; pityriasis rosea; striae rubrae |
| 38 | Klopocki 2010 / proband, family 5 | c.358A>T, p.(Lys120Ter) | nonsense | Exon 5 | SS-described | Exact height NR | Short 3rd-5th metacarpals | NR | NR | Present: oligodontia | NR | NR | Additional affected relatives described |
| 39 | Klopocki 2010 / proband, family 4 | c.532A>G,p.(Ter178GlyextTer53) | stop-loss | Exon 5 | SS-SDS | -2.4 SDS at 14 y | Short 3rd and 5th metacarpals | NR | NR | NR | NR | NR | Hypoplastic first fingernail; molecularly confirmed carrier |
| 40 | Klopocki 2010 / mother of proband, family 4 | c.532A>G,p.(Ter178GlyextTer53) | stop-loss | Exon 5 | NR | NR | Short 5th metacarpals | NR | NR | NR | NR | NR | Molecular confirmation unclear in source summary |
| 41 | Klopocki 2010 / maternal grandfather of proband, family 4 | c.532A>G,p.(Ter178GlyextTer53) | stop-loss | Exon 5 | SS-SDS | -3.6 SDS; age NR | Short 3rd-5th metacarpals | NR | NR | NR | NR | NR | Clinically affected relative; molecular testing unavailable |

**Note:** Variants are described according to HGVS nomenclature using the PTHLH reference transcript NM_198965.2. Exon and intron locations were harmonized according to this transcript and may differ from the numbering used in the original reports. Protein consequences for splice-altering variants are shown as p.? when RNA-level effects were not experimentally confirmed. Stature category was classified as SS-SDS when height was <= -2 SDS, SS-described when short stature was explicitly described in the original report without sufficient SDS-based confirmation, Non-SS when normal stature or height > -2 SDS was reported, and NR when height information was unavailable. Phenotypic features were recorded as reported in the original publications. Mammary abnormalities were recorded only when reported. NR, not reported; NA, not applicable; SDS, standard deviation score; BDE, brachydactyly type E.
